# Anti-inflammatory and antioxidant effects of hydro-alcoholic extract of *Dicliptera cuneata Nees* aerial parts

**DOI:** 10.6026/973206300191193

**Published:** 2023-12-31

**Authors:** Parepalli Suresh, VP Karthik, Durai Pandian Chamundeeswari

**Affiliations:** 1 Department of Pharmacology, SRMC & RI, Sri Ramachandra Institute of Higher Education and Research-DU, Porur, Chennai - 600116, Tamil Nadu, India

**Keywords:** *Dicliptera cuneata Nees*, acanthaceae, protein denaturation, oxidation, free radicals

## Abstract

*Dicliptera cuneata Nees* is a traditional medicinal plant but its extract or phytochemicals are less known. Therefore, it is of interest to investigate the anti-inflammatory and antioxidant effects of aerial part hydroalcoholic extract of Dicliptera cuneata Nees. Hence, we used protein denaturation assay, FRAP assay, Nitric oxide and peroxide scavenging assays methods following standard developed techniques. The hydro-alcoholic extract exhibited dose-dependent effectiveness in all the assays and showed maximum efficacy in the assays at higher doses selected. Data shows that hydroalcholic extract of *Dicliptera cuneata Nees* showed anti-inflammatory and antioxidant properties in *in vitro* settings. It should be noted that more data is needed to further develop the extract into suitable formulations.

## Background:

In recent times, plant-based compounds have garnered significant interest owing to their potential to scavenge free radicals and exhibit antioxidant activity, both of which have positive implications for human health. [1] Antioxidants are a component of food that lessen rancidity, prevent the synthesis of harmful substances, maintain nutritional value, and extend the period of storage. [2] These antioxidants defend against oxidation and the production of free radicals, preserving biomolecules like proteins, DNA, through inhibition of protein denaturation as well as fatty acids. [3] The body uses inflammation as its defense system to fend against infections, burns, poisonous chemicals, allergies, and other dangerous stimuli. A significant response to injury, illness, or devastation, inflammation manifests as heat, redness, discomfort, swelling, and abnormal biological processes. [4] Protein denaturation is a method by which its structure is distorted, or has its secondary and tertiary structures disrupted, as a result of exposure to external stimuli such as heat, strong acids or bases, organic solvents, or concentrated inorganic salts. When substrates can no longer bind to the active site, enzymes become inactive. NSAID_s_ and similar medications have a number of side effects, one of which is stomach irritation, which can lead to the formation of ulcers in the stomach. [5] Numerous plant-based extracts, including those from the foliage, bark, root systems, fruits, and seeds, have had their antioxidant as well as anti-inflammatory properties well investigated. [6] The plant *Dicliptera cuneata Nees* is a member of the acanthaceae family. Numerous researches have proven the antioxidant capacity of many Acanthaceae plants. [7] Therefore, it is of interest to assess the antioxidant capacity and anti-inflammatory properties of *Dicliptera cuneata Nees* hydro-alcholic extract.

## Materials and Methods:

## Preparation of plant extract:

We collected and authenticated the aerial parts of *Dicliptera cuneata Nees*. After being shade-dried, they were pulverised. Using the previously mentioned procedures, the powdered extract was utilised to prepare hydro alcohol extract. [8] All the chemicals used in the study were of analytical grade and obtained from Sigma Aldrich, India.

## Serial dilutions:

The extract and reference medication (acetyl salicylic acid) were serially diluted from 1000 µg/ml to 0.01 µg/ml. Each sample had a total volume of 5.0 millilitres. 2.8 ml of phosphate-buffered saline (pH 6.4) and 0.2 ml of egg albumin were used to create reaction mixtures. Following that, reaction mixtures were gently combined with 2 millilitres of extract from each separate concentration. For reference medications, which served as the study's positive controls, a similar process was followed. Distilled water was also employed as a negative control.

## Inhibition of protein denaturation:

500 µl of 1% BSA was added to 100 µl of the extract. The blend was left to incubate at 37 degrees Celsius for ten minutes. For twenty minutes, the contents were heated in a water bath at 51°C. After allowing it to reach room temperature, compare the absorbance at 660 nm with the blank. Water served as the product control and acetyl salicylic acid as the positive control. The percentage inhibition was calculated using formula below. [9]

% Inhibition = 100-{(A1-A2)/A0*100},

Where A0 is Positive Control (acetyl salicylic acid), A1 is Test Sample (extract), A2 is Negative Control (water).

## Estimation of antioxidant activity:

The antioxidant activity was analysed using Ferric ions reducing antioxidant power (FRAP assay), hydrogen peroxide and nitric oxide scavenging activity.

## FRAP *assay*:

The absorbance that results with the synthesis of Perl's Prussian blue complex when excess ferric ions (Fe3+) are used to measure the antioxidant power of samples that were initially in a state of Fe3+ (CN-)6, which later changes to Fe2+(CN-)6. Following the preparation of plant extracts in 0.75 ml of distilled water at different concentrations, 1.25 ml of sodium phosphate buffer (0.2 M) a nd 1.25 ml of potassium hexacyanoferrate (III) (1%), were combined. After 20 minutes of being kept at 50°C in the incubator, 1.25 millilitres of 10% trichloroacetic acid were added to the ingredients in order to acidify it. The absorbance of this solution was then measured at a wavelength of 600 nm using spectrophotometry after mixing 0.5 ml of Iron (III) chloride (0.1%). Employing the formula below, the FRAP value of extract was determined based on their absorbance readings in comparison to the standard's absorbance reading. [10]

FRAP Value (µmol/L)=(Absorbance at 593nm of test sample reaction mixture)/(Absorbance at 593nm of Ferrous ion Standard reaction mixture)xFerrous ion Standard concentration

## Hydrogen peroxide scavenging *assay*:

In phosphate buffer (pH 7.4), a 40 mM hydrogen peroxide concentration was developed. A sample containing 10 mg/10µl was dissolved in to 0.6 millilitres of H2O2 solution. Phosphate buffer was added to get the volume up to 3 ml. At 230 nm, the reaction mixture's absorbance was measured. Phosphate buffer without H2O2 existed in the blank solution. The percentage of H2O2 scavenging exhibited with plant extract was computed as

*% scavenged hydrogen peroxide* = (A0-A1) x 100 ÷ A0

Where A0 - Absorbance of control, A1- Absorbance in the presence of extract [11]

## Nitric oxide scavenging *assay*:

For this quantification, plant extract was dissolved in distilled water. For three hours, tubes containing sodium nitroprusside (5 mM) in standard phosphate buffer saline (0.025 m, pH 7.4) were incubated at 29°C with varying concentrations (100-400 µg/ml) of extract. The control experiment was carried out in the same way but without the test chemicals and with a similar volume of buffer. The incubated samples were diluted with 1 millilitre of Griess reagents after three hours. Using a spectrophotometer, the absorbance of the colour that resulted from diazotizing nitrite with sulfanilamide and coupling it with napthylethylenediamine hydrochloride was measured at 550 nm. The same process was carried out using ascorbic acid, which was standard to extract. By using a formula and plot graph in comparison to a standard, the percentage inhibition was calculated. [12]

## Results:

## Anti-inflammatory activity:

The *Dicliptera cuneata Nees* hydroalcoholic extract anti-inflammatory properties were assessed using the egg albumin denaturation method. With extract, the inhibition rate was seen in a dose-dependent manner. At the higher doses, extracts showed stronger inhibition (Table 1).

## Antioxidant activity:

The reducing power (absorbance at 600 nm), nitric oxide, and hydrogen peroxide scavenging activity (percent decolorization) were used in this investigation to assess the antioxidant properties of the *Dicliptera cuneata Nees* hydroalcoholic extract. The FRAP was increasing with the dose escalation of the extract. The maximum FRAP value was 590 at dose of 1000 µg/ml of Dicliptera cuneata Nees hydroalcoholic extract (Table 2).

The nitric oxide scavenging capacity of *Dicliptera cuneata Nees* hydro-alcoholic extract was compared with ascorbic acid. The nitric oxide scavenging capacity was shown in a dose dependent manner by the extract and was maximum 88% at 1000 µg/mL (Table 3). The scavenging effects of *Dicliptera cuneata Nees* hydroalcoholic extract on the H2O2 radicals expressed by percentage is shown in Table 4. The hydrogen peroxide H2O2 scavenging test showed that the extract exhibited the best antioxidant effect by producing 64.5% scavenging effect at 1000 µg/mL

## Discussion:

Based on the results of the FRAP test, the *Dicliptera cuneata Nees* hydroalcoholic extract was found to have the most potent iron-reducing power when it was tested for antiradical activity. However, the H2O2 trapping test investigation of the antioxidant activity revealed that the extract has the maximum H2O2 and nitric oxide radical-inhibiting action. The free radicals are known to cause tissue or cellular damage, [13] which can be attenuated by this extract. Protein denaturation occurs through a mechanism that is unexpected and involves changes to hydrophobic, disulfide, and electrostatic hydrogen bonds. [14] In inflammatory diseases such rheumatoid arthritis, cancer, [15] neurodegeneration [16-17] and diabetes, [18] denaturation of proteins results in the creation of autoantigens. Therefore, it is possible to reduce inflammatory activity by inhibiting protein denaturation. [19] Acetylsalicylic acid (NSAID) was employed as reference medications in the current investigation. By inhibiting the activity of the cyclooxygenase enzyme, NSAID_s_ reduce inflammation. On the other hand, ulceration, bleeding, perforation, and blockage are adverse outcomes of these medications. [20] The *Dicliptera cuneata Nees* hydroalcoholic extract exhibited potent inhibition of protein denaturation which may be used as replacement to acetylsalicylic acid after thorough research.

In comparison, the plants of the same family showed antioxidant properties in previous research. Using the DPPH assay method, a study assessed the antioxidant properties of leaf extract from *Phaulopsis fascisepala*, an acanthaceae plant. This investigation found that the methanolic extract had a 53.4% DPPH scavenging activity at a dosage of 0.3 mg, suggesting that it might be a moderate antioxidant. [21] Another study used the DPPH assay method to assess antioxidant activity in aqueous acetone extract (80%) from leaves of acanthaceae plants, such as *Blepharis lineariifolia*, *Dicliptera verticillata*, *Dyschoriste perrottetii*, *Hygrophila auriculata*, *Lepidagathis anobrya*, and *Nelsonia canescens*. [22] Because of the anti-inflammatory and antioxidant properties, the commercially available medicinal plant extracts are utilised as herbal remedies in several nations. However, their exact mode of action remains unclear. [12] Furthermore, the type of biologically active ingredient included in the extract as well as phenolic chemicals like flavonoids and polyphenols may help to explain the actions of the extract. [23]

## Conclusion:

Compared to reference medications (NSAID_s_, such as acetylsalicylic acid), the *Dicliptera cuneata Nees* hydroalcoholic extract exhibits substantially more effective anti-inflammatory properties against the denaturation of egg albumin. This also possessed potent antioxidant property on iron and hydrogen peroxide free radicals. It should be noted that additional *in vivo* and *in vitro* data is needed to confirm the extract's anti-inflammatory properties.

## Figures and Tables

**Table 1:** Anti-inflammatory activity of *Dicliptera cuneata Nees* hydroalcoholic extract by Inhibition of Protein Denaturation

**S. No**	**Concentration (µg/ml)**	**Sample**	**Positive Control**	**% inhibition**
		**O.D**	**O.D**	
1	20	0.687	0.665	44.51
2	40	0.613	0.632	53.32
3	60	0.511	0.532	63.72
4	80	0.445	0.515	75.33
5	100	0.389	0.482	85.26
Negative Control O.D: 0.318

**Table 2:** FRAP of *Dicliptera cuneata Nees* hydroalcoholic extract

**S. No.**	**Concentration (µg/ml)**	**0^th^ minute**	**4^th^ minute**	**FRAP Value**
1	200	0.15	0.256	530
2	400	0.207	0.316	545
3	600	0.288	0.4	560
4	800	0.416	0.53	570
5	1000	0.53	0.648	590

**Table 3:** Nitric oxide scavenging activity of *Dicliptera cuneata Nees* hydroalcoholic extract

**Sl No.**	**Concentration (µg/ml)**	**O.D.**	**NO % Inhibition**
1	200	0.248	73.13
2	400	0.214	76.81
3	600	0.18	80.49
4	800	0.146	84.18
5	1000	0.112	87.86
Control OD value is 1.021

**Table 4:** Hydrogen peroxide scavenging activity of *Dicliptera cuneata Nees* hydroalcoholic extract

**S. No**	**CONCENTRATION (µg/mL)**	**O.D VALUE**	**% SCAVENGED HYDROGEN PEROXIDE**
1	200	0.455	1.515
2	400	0.385	16.66
3	600	0.309	33.11
4	800	0.24	48.05
5	1000	0.164	64.5
Control OD value is 0.462
